# Tree Architecture and Structural Complexity in Mountain Forests of the Annapurna Region, Himalaya

**DOI:** 10.1002/ece3.71341

**Published:** 2025-04-27

**Authors:** Smita Das, Prakash Basnet, Dominik Seidel, Alexander Röll, Martin Ehbrecht, Dirk Hölscher

**Affiliations:** ^1^ Tropical Silviculture and Forest Ecology University of Göttingen Gottingen Germany; ^2^ Department for Spatial Structures and Digitization of Forests University of Göttingen Gottingen Germany; ^3^ Centre of Biodiversity and Sustainable Land Use University of Göttingen Gottingen Germany; ^4^ Horticultural Sciences University of Bonn, Institute for Crop Science and Resource Conservation Bonn Germany; ^5^ Department of Silviculture and Forest Ecology of the Temperate Zones University of Göttingen Gottingen Germany

**Keywords:** box‐dimension, elevation, precipitation, structural complexity, tree architecture

## Abstract

Mountain ranges comprise heterogeneous environments and high plant diversity, but little is known about the architecture and structural complexity of trees in mountain forests. We studied the relationship between tree architecture, environmental conditions, and tree structural complexity in forests of the Annapurna region in the Himalaya. We further asked whether and how tree structural complexity translates into forest stand structural complexity. The study covers 546 trees on 14 undisturbed study plots across wide ranges of elevation (1300 to 3400 m asl.) and annual precipitation (1180 to 3600 mm yr.^−1^). They were assessed by ground‐based mobile laser scanning. We found that tree structural complexity, expressed as box‐dimension (*D*_*b*_), was lowest for the needle‐leaved *Pinus wallichiana* and highest for the broad‐leaved *Daphniphyllum himalense*. A high share of the variation in *D*_*b*_ was explained by tree architecture. In multivariate models, tree height, crown radius, and crown length explained more than 60% of the observed variation in *D*_*b*_. Stem density of the plot accounted for 19% of the variation in *D*_*b*_, and there was no influence of tree diversity. Precipitation explained l3% of the observed variation in tree *D*_*b*_, but elevation and slope did not have significant influences. As expected, tree height decreased with increasing elevation, but small trees often had relatively high *D*_*b*_ values. The standard deviation of tree‐level *D*_*b*_ within a plot explained 47% of the variation in stand‐level structural complexity among plots, surpassing the maximum tree‐level *D*_*b*_. This suggests that both the sole removal of small or large trees would reduce the stand‐level complexity by 36%. We conclude that in the Himalayan forests, species identity and tree architecture play a significant role in determining tree structural complexity, while environmental factors have a smaller role. Furthermore, structural variation among the trees within a plot plays a crucial role for the structural complexity at the stand level.

## Graphical abstract

Tree architecture and structural complexity were studied in mountain forests of the Himalaya. We assessed 546 trees belonging to six species by mobile laser scanning. The change in tree structural complexity with elevation was less pronounced than the decline in tree height. Species identity and tree architecture played a significant role in determining tree structural complexity. Structural variation among the trees determines the structural complexity at the stand level.

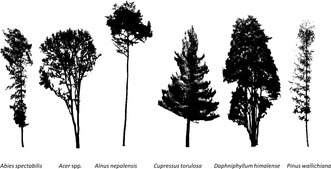

## 1 Introduction

Mountain ranges comprise huge abiotic heterogeneity and harbor a high diversity in plant species including trees (Cai et al. 2023; Keil and Chase 2019). Environmental factors such as elevation, precipitation, and slope steepness affect tree species occurrence, tree architecture, and structure (Cheng et al. 2023; Homeier et al. 2010). An architecture‐based approach may help to better understand variations among and within species and their interactions with the environment and neighboring trees (Laurans et al. 2024). At stand level, structural complexity is strongly controlled by environmental variables and forest management (Ehbrecht et al. 2021); increasing complexity enhances forest functions including productivity (Ray et al. 2023; Soto et al. 2024) and habitat provisioning (Zemp et al. 2023). In general, the relationship between tree structural complexity and stand‐level complexity has rarely been explored and points to the importance of variance and maxima of tree structural complexity in a stand (Seidel et al. 2019b). In particular, no such information is available for mountain forests in the Himalaya, where climate change and socio‐economic dynamics drive changes in forest structures (McGunnigle et al. 2025; Negi et al. 2022). This study explores the relationships between environment, tree architecture, tree, and stand structure complexity for undisturbed forests in the Annapurna range in order to create a baseline for a better understanding of forest dynamics.

Tree architecture refers to the structural organization encompassing the arrangement of its branches, leaves, and other components (Hollender and Dardick 2015; Laurans et al. 2024). Tree architecture varies between and within species, from pole‐like structures to expansive, multi‐stem trees (Beech et al. 2017). The sources of variability are due to interspecific variability, intraspecific genotypic diversity, and phenotypic plasticity (Laurans et al. 2024). Trees demonstrate high plasticity, adapting their structures in response to environmental stimuli (Tomlinson 1983). Environmental factors such as wind, slope, light, and water were shown to influence tree architecture (Dorji et al. 2021; Magnin et al. 2017; Seidel et al. 2019c; Van de Peer et al. 2017; Wang et al. 2023). It is further influenced by stand density and species diversity (Juchheim et al. 2020). Tree architecture is key in determining the overall tree structural complexity (Seidel et al. 2019a).

Structural complexity is defined as the aggregate of dimensional, architectural, and distributional characteristics of all plant individuals in a defined area at a given time (Seidel et al. 2019b). It is necessary to aggregate information collected at the individual plant level to gain insight into the driving forces behind population or community structure and dynamics (Laurans et al. 2024; Salguero‐Gómez et al. 2018; Shipley 2010). Structurally complex trees may create complex forest stand structures essential for forest vitality (Heidenreich and Seidel 2022), ecosystem resilience, biodiversity conservation, and climate regulation (Coverdale and Davies 2023; Hansen et al. 1991; Messier et al. 2019; Rajaonarimalala et al. 2024; Zenner and Hibbs 2000).

Traditional methods of studying tree architecture involved manual measurements of tree height, diameter at breast height (DBH), and crown structure (Ehbrecht et al. 2017; Iizuka et al. 2018; Owen and Lines 2024). Advancements in light detection and ranging (LiDAR) technologies facilitated measurements of the three‐dimensional architecture of trees (LaRue et al. 2020; Martin‐Ducup et al. 2020; Rieder et al. 2024; Wang et al. 2019). Based on the fractal analysis approach proposed by Mandelbrot (1977), a comprehensive metric for assessing the structural complexity of trees, the box‐dimension (*D*_*b*_), was developed (Seidel 2018). The process of calculating box‐dimension involves using boxes or voxels of different sizes to encompass all 3D points from individual trees that were captured using a laser scanner (Seidel 2018). The box‐dimension is a scale‐independent metric for evaluating tree architecture that also describes the entire aboveground structural complexity of the tree (Seidel and Böttger 2023).

Structural complexity at tree‐level, with its potential relationships to different tree functions, is not yet fully understood (Laurans et al. 2024). At stand level, multiple positive effects have been observed (Ray et al. 2023; Soto et al. 2024; Zemp et al. 2023), and management for complexity has become an important paradigm for European and North American forestry (Seidel et al. 2019b). To our best knowledge, so far only a single study in a broad‐leaved forest in Central Europe explored the relationship between tree‐level and stand‐level complexity. It suggests that stand‐level complexity is largely determined by the complexity of the most complex‐structured tree individual (Seidel et al. 2019b). Since the study covered only one forest region with relatively uniform environmental conditions, there is a need for further exploration and validation of these relationships. For the Annapurna range in the Himalaya of Nepal, Basnet et al. (2025) assessed stand structural complexity on 69 plots across highly variable environmental conditions. They found a high predictive power of variables such as the number of trees, tree height, tree species diversity, and disturbance; environmental variables played a minor role (Basnet et al. 2025). This data set provides an excellent opportunity to further test the relationship between tree‐level and stand‐level complexity.

Our study is mainly at the tree level and explores the relationships between environment, tree architecture, and tree structure complexity for undisturbed forests in the Annapurna range. It may create a baseline for better monitoring and understanding possible tree and forest dynamics in mountain forests of the Himalaya. We hypothesized that (i) tree species differ in tree structural complexity, (ii) tree architecture controls tree structural complexity, (iii) tree structural complexity decreases with increasing elevation and decreasing precipitation, and (iv) tree structural complexity controls stand structural complexity.

## 2 Methods

### 2.1 Study Region and Trees

**FIGURE 1:**
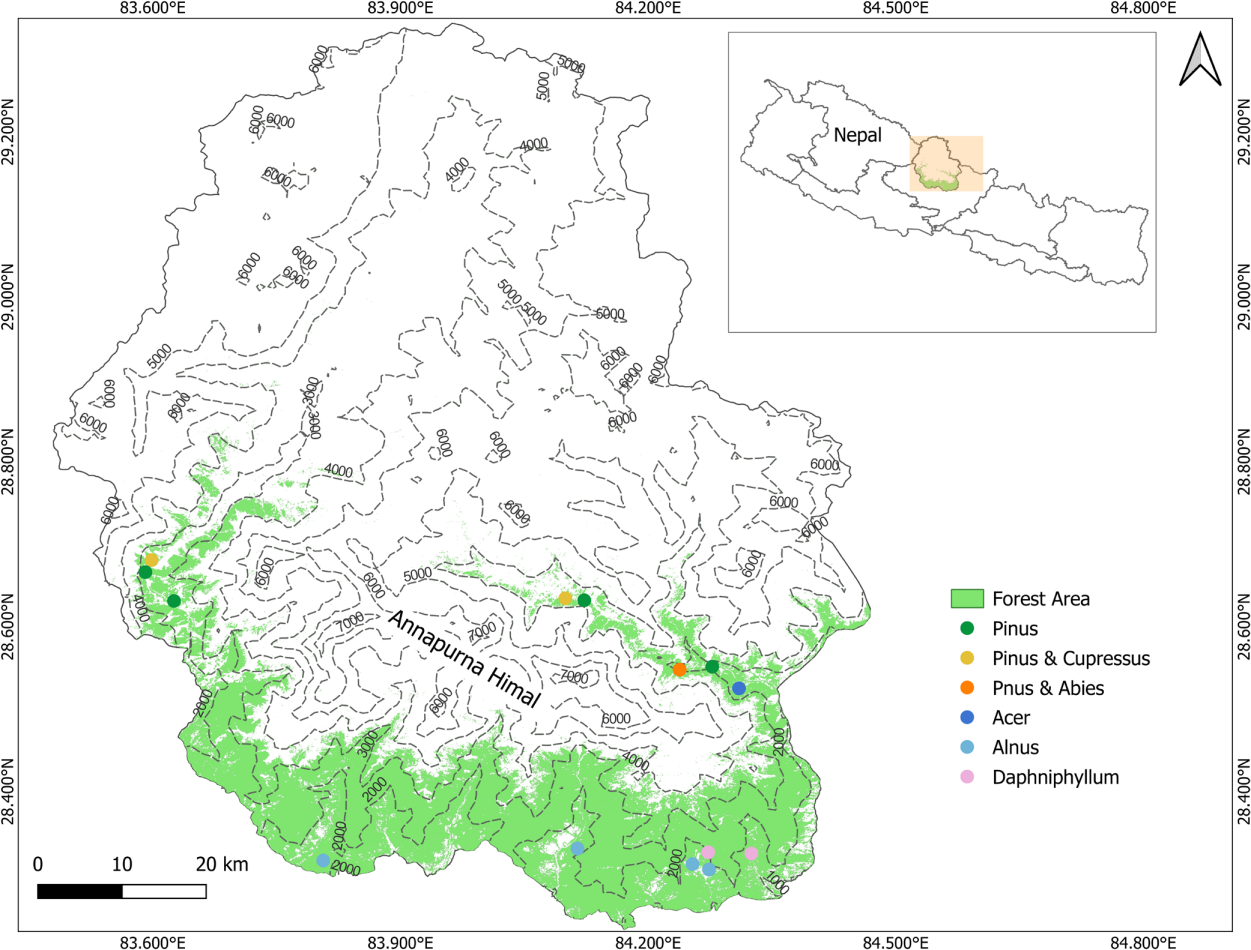
The Annapurna Conservation Area in Nepal, its forest area (green areas, Potapov et al. 2021), our 14 study plots (dots), and their dominant tree species (different colors). The light gray area encompasses shrubland, grassland, barren land, rocks, and glaciers.

The study was conducted in the forests of the Annapurna Conservation Area (ACA), which encompasses the Annapurna range, located in the central Himalayan region of Nepal (Figure 1). It covers 7629 km^2^ and is located between 83°34′ to 84°25′ E longitude and 28°15′ to 28°50′N latitude. The ACA falls into the IUCN management category VI, which allows sustainable use of natural resources. The area comprises complex topography, pronounced climatic gradients, and a high plant species diversity (Paudel et al. 2012). Elevation above sea level ranges from approx. 1000 m to more than 8000 m in the Annapurna Himal. The mean annual air temperature changes substantially along the large altitudinal gradient, with 19.8°C in the lower parts (~1000 m), −0.92°C at the tree line (4200 m, Shrestha et al. 2007) and −12°C at the high mountains (7000 m) CHELSA data V2.1(1981–2010), (Karger et al. 2021).

The Great Himalaya can be divided into two climatic regions: the Cis‐Himalayan region to the south and the Trans‐Himalayan region to the north. The mountains act as a barrier to monsoon winds, leading to significant differences in precipitation between the regions (Andermann et al. 2011). The Cis‐Himalayan region, i.e., the windward side, receives heavy rainfall, while the Trans‐Himalayan region, i.e., the leeward side, is much drier (DFRS 2015). In Nepal's Annapurna Conservation Area, this contrast is evident, with the windward side receiving up to 5000 mm of rainfall annually in areas like Lumle, Sikles, and Bhujung while the northern, leeward side, in areas such as Mustang and Manang, records as little as 194 mm per year (CHELSA data V2.1 (1981–2010), Karger et al. 2021). The area's climate is strongly influenced by the monsoon, which brings heavy rains from June to September, followed by dry winter months (Dangi et al. 2018). The geological composition comprises distinct formations such as gneiss, schist, limestone, and shale of different ages and characteristics (DFRS 2015). Forests cover 27% of the total area of the Annapurna Conservation Area (Potapov et al. 2021) (Figure 1). Tree species comprise broad‐leaved species such as *Alnus nepalensis, Acer campbelli, Betula utilis, Castanopsis indica, Daphniphyllum himalense, Rhododendron* spp., *Schima wallichii*, and needle‐leaved species such as *Abies spectabilis, Cupressus torulosa, Juniper indicia, Pinus roxburghii*, *Pinus wallichiana* and *Tsuga dumosa* (Paudel et al. 2012).

**FIGURE 2:**
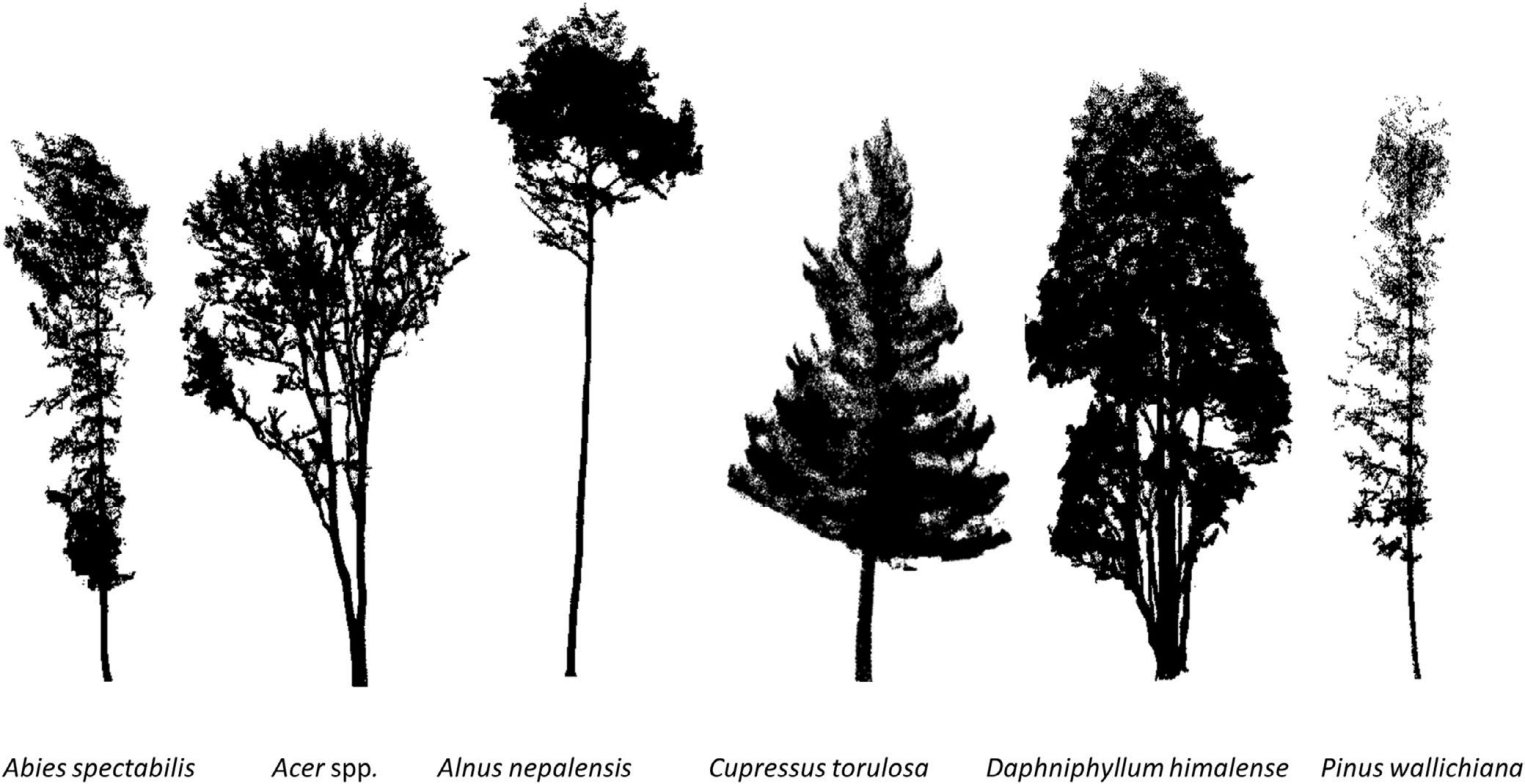
Examples of tree shapes for each of the six studied species as derived from mobile laser scanning. The species *Abies spectabilis*, *Cupressus torulosa* , and *Pinus wallichiana* are needle‐leaved; Acer spp., *Alnus nepalensis* , and *Daphniphyllum himalense* are broad‐leaved.

The studied tree species are shown in Figure 2.

### 2.2 Study Design

Our study mainly focused on tree‐level complexity (*n* = 546 trees). It was conducted on 14 undisturbed plots distributed in the forests surrounding the Annapurna Himal (Figure 1), which are a subset of the 69 plots studied by Basnet et al. (2025) who focused on stand‐level structural complexity. The 69 plots were placed to match with GEDI (Global Ecosystem Dynamics Investigation) observations, a spaceborne LiDAR mission that generates global‐scale forest maps (Duncanson et al. 2022). The study plots were circular, with a diameter of 25 m. The 14 plots were selected to represent a baseline of largely undisturbed forest, cover wide environmental gradients (elevation: 1300 to 3400 m asl.; and mean annual precipitation: 1180 to 3600 mm yr.^−1^), and to include broad‐leaved forest (7 plots) and needle‐leaved forest (7 plots). 13 plots showed no signs of disturbance, while one plot displayed evidence of former cattle grazing. The trees located in those plots belong to the needle‐leaved species *Abies spectabilis, Cupressus torulosa, Pinus wallichiana, and* the broad‐leaved species *Acer* spp., *Alnus nepalensis, and Daphniphyllum himalense* (Figure 2). At the time of the study, all trees were leafed except for *Acer* spp. Field data were collected between September and December 2021.

### 2.3 Environmental Variables

Mean annual precipitation (mm year ^−1^) was extracted from CHELSA time series data version 2.1 (1981–2010) (Karger et al. 2021), which is the most up‐to‐date database for regional studies and suited for mountainous areas. Elevation data were downloaded from the ASTER Global Digital Elevation Model (DEM) V003 (NASA/METI/AIST/Japan Spacesystems and U.S./Japan ASTER Science Team 2019). Aspect data were derived from the DEM using the Raster terrain analysis plugin in QGIS V3.22.5 (QGIS Development Team 2022). Aspect data (in degrees) was transformed to northness/eastness: northness = cos (aspect) and eastness = sin (aspect), where aspect must be expressed in radians (radian = degree * pi/180). Northness ranges from values close to 1 if the aspect is mostly northward and close to −1 if the aspect is southward. Similarly, an eastness value close to 1 represents east‐facing slopes and close to −1 west‐facing slopes (Cursach et al. 2020; Roberts 1986). Slope was measured in the field, aiming to the plot center once from the downward side of the plot boundary, with an Abney level (Sokkia No. 8047–4, Japan). A summary of environmental characteristics across the 14 study plots can be found in Table A1.

### 2.4 Mobile Laser Scanning

We used the handheld mobile laser scanner ZEB‐Horizon (*GS‐610090*, GeoSLAM Ltd., Nottingham, United Kingdom, 2020). The device was carried through the stand while capturing objects within a maximum range of 100 m using 16 time‐of‐flight LiDAR sensors. The wavelength of the laser is 903 nm, and it scans 300,000 points per second with a relative accuracy of up to 6 mm. The point density from our field data ranges from approx. 27,000 to 53,000 points per m^2^, with an average of 45,000 points per m^2^. The scanner was held at breast height while walking a pre‐defined, closed path in a zigzag pattern to cover all trees in 25 m diameter circular plots to capture 3D point cloud data of the forest structure.

### 2.5 Point Cloud Processing

The raw scanned point cloud data were downloaded and processed with the GeoSLAM Hub V6.1 software (GeoSLAM Ltd., Nottingham, United Kingdom) to convert the raw data into text format files. The text files were then imported into LiDAR 360 V5.0 software (Green Valley International Ltd., CA, USA) for post‐processing. Based on the LiDAR 360 V5.0 user guide (https://greenvalleyintl.com/), post‐processing steps were applied, which included outlier removal, ground normalization to remove the topographic relief effect, and ground point classification to classify ground and non‐ground points. Subsequently, a subsampling process was applied for homogeneous point cloud density by using a minimum point spacing (0.01 m) and a noise filter (0.1 m radius) (Dorji et al. 2021). Following that, a slope correction procedure was applied using the formula (*r*_corr = *r* * cos(*α*)), where *r* is the radius of the plot in sloped terrain, and *α* is the slope angle (Kleinn et al. 2011). Subsequently, we automatically segmented each tree within the refined and clipped point clouds of each plot, generating individual tree data. However, dense vegetation and overlapping tree crowns led to inaccuracies in representing individual trees. To address this, we used open‐source CloudCompare V2.13 beta software (https://www.danielgm.net/cc/) to manually segment and improve these individual point clouds. All trees with their stem base and tree crown located inside the plot border and with a tree height of at least 3 m were considered sample trees in our study.

### 2.6 Structural Complexity (*D*_*b*_) on Tree and Stand Level

The structural complexity of the 546 trees was determined by assessing their box‐dimension (*D*_*b*_). *D*_*b*_ is calculated by using a fractal analysis approach to measure objects' structural complexity (Seidel et al. 2019b). The process of calculating the box‐dimension involves using boxes of varying sizes to encompass all 3D points of individual trees (Seidel 2018). The same procedure was applied to calculate plot‐level *D*_*b*_ using the merged point clouds of all trees present on a respective plot (Basnet et al. 2025). Theoretically, *D*_*b*_ for natural three‐dimensional objects ranges from one to three (Mandelbrot 1977). A *D*_*b*_ value of 1 is possible for cylindrical, pole‐like objects, while a *D*_*b*_ value of 3 corresponds to a solid cube. We used an algorithm written in Mathematica software V14.0 (Wolfram Research, Champaign, USA) (Seidel 2018). For trees, the theoretical maximum *D*_*b*_ value has been indicated to be significantly lower than 2.72 (Seidel et al. 2019c).

### 2.7 Tree Architecture

All tree architecture attributes as shown in Table 1 were calculated using the algorithms written in Mathematica software V14.0 (Wolfram Research, Champaign, USA). The algorithm allowed for the parameterization of various structural attributes of the tree's crown and stem using the XYZ data of each tree. This was achieved by transforming the single tree point cloud into a voxel model (Seidel et al. 2011).

**TABLE 1:** Structural attributes at single tree level.

Variable	Abbreviation
Total tree height (m)	TTH
Diameter at breast height (cm)	DBH
Crown base height (m)	CBH
Crown surface area (m^2^)	CSA
Maximum crown area in a single height layer (m^2^)	Max_crownarea_
Height of maximum crown area (m^2^)	H_maxarea_
Mean crown radius (m)	MCR
Crown volume (m^3^)	Crown volume
Crown length (m)	CL
Crown asymmetry (m)	Crown asymmetry

The individual trees' total height (TTH) was calculated based on the distance between a tree's lowest and highest points. Further, the diameter at breast height (DBH) at 1.3 m above ground was measured for all trees. For multi‐stem tree species, such as *Daphniphyllum himalense*, when multiple stems were separated below 1.3 m, the DBH of each stem was measured individually. Following the quadratic sum method described by Magarik et al. (2020), each stem's diameter was squared, summed, and the square root of the total was taken to yield a single DBH value. This approach was employed due to the difficulty in distinguishing individual crowns for each stem as their crowns were interrelated. Thus, this approach considered the multi‐stem configuration as a single tree with a unified crown structure. In contrast, for Acer spp., most multi‐stemmed trees exhibited stem separation above 1.3 m, with only three exceptions in our study where separation occurred below this height. In this case, we were able to easily separate and measure each stem along with its respective crown individually. Further, the automated process inaccurately measured the diameter of multi‐stem and small trees. As a result, manual DBH measurements were carried out in CloudCompare for those trees. This process entailed selecting two opposing points on a cross‐sectional disc from the point cloud at a tree height of 1.3 m. Additionally, we calculated crown base height (CBH), which is the height of the lowest leaf‐bearing branch up to the ground. Further, we calculated the crown surface area (CSA) as the surface area of the convex hull polygon of the tree crown, measured from the crown base height to the top (Juchheim et al. 2017; Seidel et al. 2019a). To determine the maximum crown projection area (Max_crownarea_), convex hull polygons were generated around all scan points in a vertical layer (10 cm thickness), and the area of the largest polygon and its corresponding height (H_maxarea_) (Seidel et al. 2015). In the case of the mean crown radius (MCR); see (Seidel et al. 2015) taken as the mean of 360 directional measurements of crown radius. Furthermore, crown volume was calculated based on Metz et al. (2013). Further, crown length (CL) was calculated by taking the difference between TTH and CBH. Crown asymmetry, a measure of relative crown plasticity, was estimated by measuring the horizontal distance between the center of the crown and the height of the maximum crown projection area.

### 2.8 Quantitative Structure Model (QSM)

**FIGURE A1:**
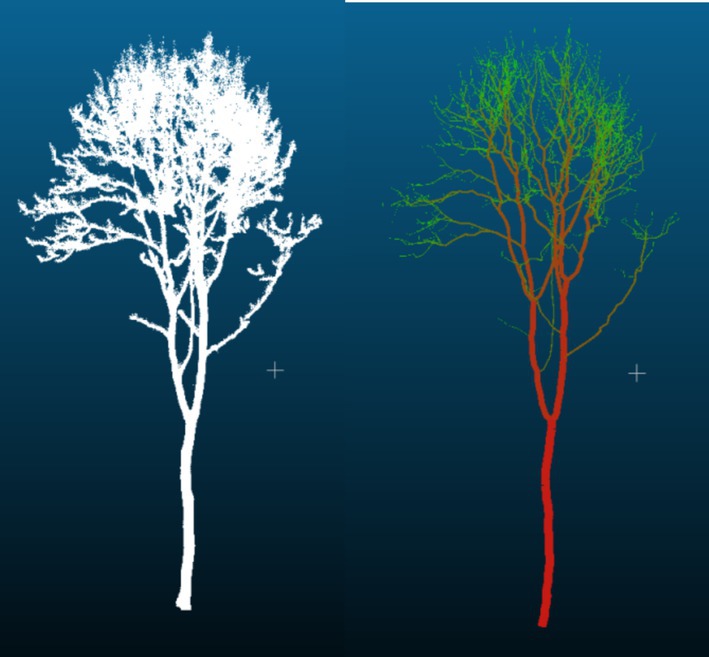
Example of a point cloud of an *Acer* tree 15 m in height (left) and the corresponding quantitative structure model (right) with cylinder formation from base to tip of the tree.

Quantitative Structure Models (QSMs) are topologically ordered cylinder models of trees that cover the complete branching structure from the base of the stem up to all tips (Hackenberg and Bontemps 2023). This method provides the aboveground volume of the tree by formation of the stems into cylinders along with all order branches but excludes foliage, fruits, and flowers (Demol et al. 2022) (an example of the QSM of one tree is shown in Figure A1). Based on the QSM approach, the software CompuTree V5.0 with the SimpleForest plugin V5.1.3 (Hackenberg et al. 2021) calculated the aboveground tree volume from its point cloud. Each tree model was built with the same parameter settings: voxel size (0.020 m), clustering range (0.90 m), for fitting a geometry minimum point needed (2 points), and for allometric correction, a minimum number of measurements needed for fitting was set to value 5 (Hackenberg et al. 2015). The total aboveground volume of each tree was calculated by summing up the volumes of the stem and all order branches. Aboveground biomass (AGB, kg) was calculated by multiplying the aboveground total volume by species‐specific wood density values (kg m^−3^) as provided for the studied tree species (DFRS 2015).

### 2.9 Tree Interaction

Shannon Diversity Index (H) (Shannon 1948), species richness, and plot‐level stem density were computed as indicators of tree interaction.

### 2.10 Statistical Analysis

We used Box‐and‐Whisker plots to explore the central tendency and variability of total tree height (TTH), diameter at breast height (DBH), and box‐dimension (*D*_*b*_) values across tree species. Given the unequal sample sizes and variances among the species, we employed Welch's *t*‐test to evaluate significant differences in *D*_*b*_ and structural characteristics among the species. Statistical significance was established at *p* < 0.05.

When examining the relationship between each tree's *D*_*b*_ and its architectural attributes, we conducted a single‐factor analysis using Spearman's rank‐order correlation coefficient (r). Following the initial analysis of our findings, we conducted a multiple linear regression to assess the overall impact of different combinations of tree architectural characteristics on *D*_*b*_. Multicollinearity among the independent variables was assessed by calculating variance inflation factor (VIF) values, with all VIFs maintained below 5 (Kyriazos and Poga 2023). The best model was determined through a stepwise variable selection process to minimize the Akaike Information Criterion (AIC) and achieve a low root mean square error (RMSE). We also performed a simple linear regression to assess the relationship between an individual tree's sum, maximum, and standard deviation of *D*_*b*_ within a plot and the overall stand‐level *D*_*b*_. Model validation was carried out by ensuring the satisfaction of the necessary assumptions of linearity, homoscedasticity, and normality of residuals, and applying data transformations as required.

Linear mixed‐effects model (R package lme4, V1.1–35.4; Bates et al. 2015) was applied to predict *D*_*b*_ using the Shannon Index, species richness, stem density, and the environmental variables (precipitation, elevation, and slope) as fixed factors and plot as a random factor. In the mixed‐effects model, predictor variables were standardized using the scale function, by centering predictors to have a mean of zero and scaling them to have a standard deviation of one. This standardization allows for the comparison of effect sizes across the estimated coefficients. We checked for spatial dependency of the residuals using Moran's *I* test. Model residuals were found to be not spatially auto‐correlated (observed Moran ´s *I* = −0.001, *p* = 0.86, R‐package ape V5.0; Paradis et al. 2024). All statistical analyses were performed using R 4.4.1 (R Core Team 2024).

## 3 Results

### 3.1 Height, DBH, and *D*_*b*_ per Species

**FIGURE 3:**
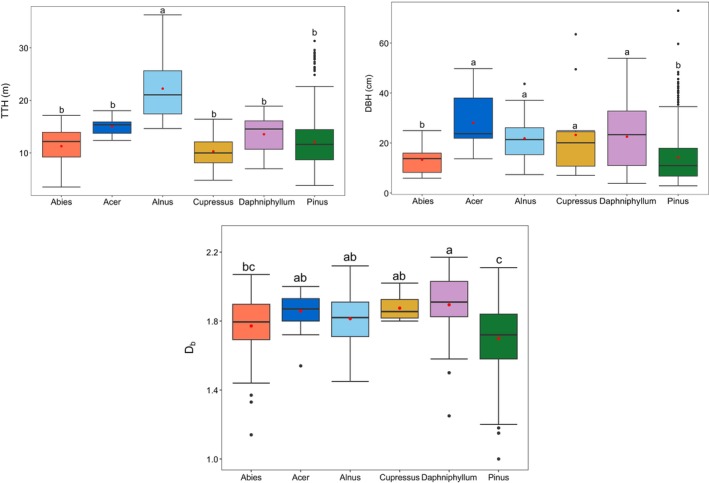
Total tree height (TTH), diameter at breast height (DBH), and tree structural complexity as box‐dimension (*D*_*b*_) by species. *Abies* (*n* = 40), *Acer* (*n* = 17), *Alnus* (*n* = 65), *Cupressus* (*n* = 12), *Daphniphyllum* (*n* = 55), *Pinus* (*n* = 357) trees per species. Different letters indicate significant differences in mean at *p* < 0.05 using Welch's *t*‐test.

**FIGURE A2:**
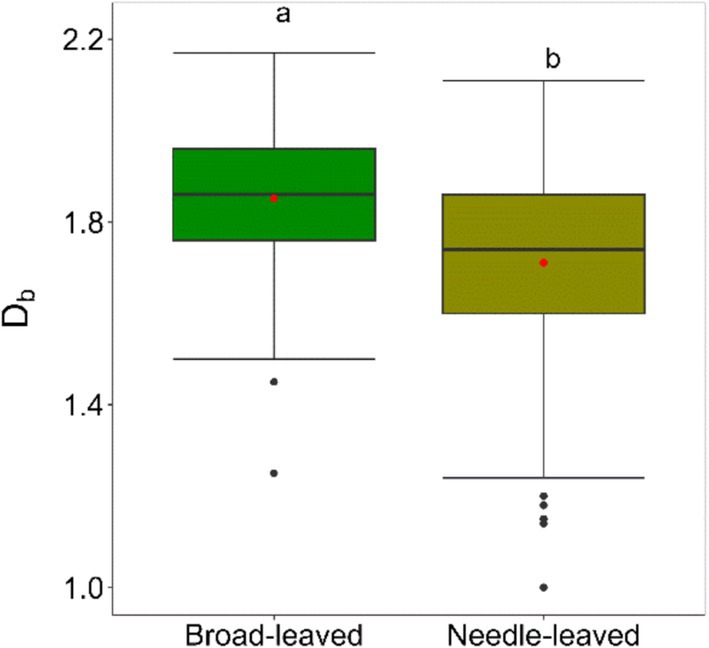
Box‐dimension (D_b_) for all broad‐leaved (*n* = 137) and needle‐leaved (*n* = 409) trees. *p* < 0.05.

Among the 546 sample trees, the tallest reached 36.28 m ( *Alnus nepalensis* ), and DBH ranged from 3 cm to 73 cm. *Alnus nepalensis* had the largest mean tree heights, while *Cupressus torulosa* had the smallest (Figure 3). Acer spp. had the largest mean DBH, while *Abies spectabilis* had the smallest. Among the 55 sampled *Daphniphyllum himalense* trees, 19 trees exhibited multiple stem separation below a height of 1.3 m, with stem counts varying between 2 to 8 and DBH ranging from 11 to 54 cm. As for height and DBH, mean box‐dimension (*D*_*b*_) values varied significantly among species and showed high variability within species. At the species level, *Pinus wallichiana* had the lowest mean *D*_*b*_ (1.70) and *Daphniphyllum himalense* the highest (1.89). Needle‐leaved trees combined had a lower average *D*_*b*_ (1.71 ± 0.01) than all broad‐leaved trees combined (1.85 ± 0.01) (Figure A2).

### 3.2 *D*_*b*_ and Architecture

**FIGURE 4:**
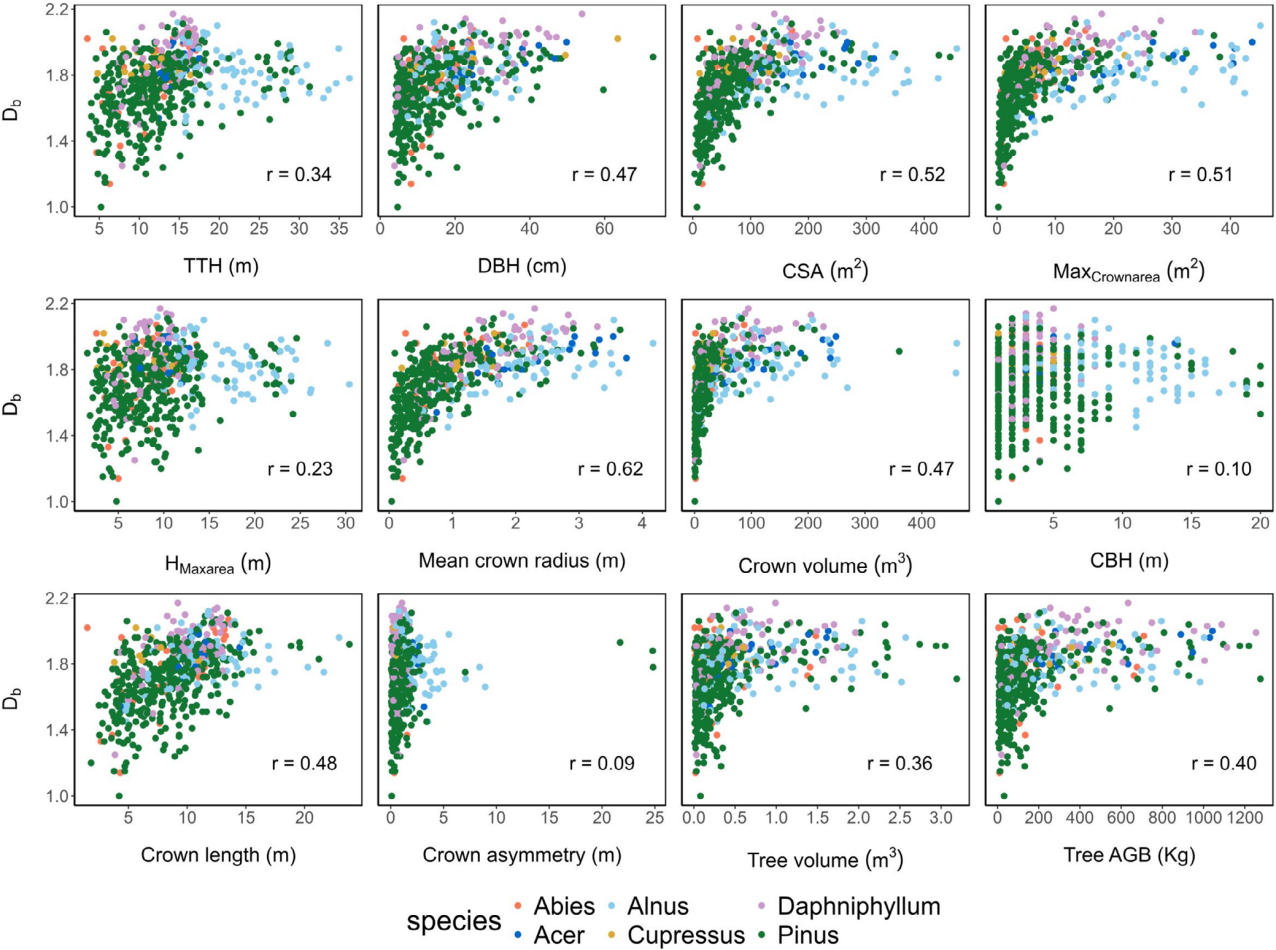
Relationships between tree structural complexity (box‐dimension D_b_) and architectural characteristics and biomass for *n* = 546 sample trees. Different color dots indicate different tree species. TTH—Total tree height, DBH—Diameter at breast height, CBH—Crown base height, H_maxarea_)—Height of maximum crown projection area, Max_crownarea_—Maximum crown area, CSA—Crown surface area, AGB—Aboveground biomass. All depicted relationships (i.e., r values) are significant at *p* < 0.05.

*D*_*b*_ ranged from 1.0 to 2.17 for the studied trees (*n* = 546). *D*_*b*_ had a significant relationship with several tree architectural characteristics (Figure 4), wherein the strength of the relationship, as expressed by correlation values, varied among characteristics. As such, crown asymmetry had a very low correlation (*r* = 0.09) while mean crown radius was strongly related to *D*_*b*_ (*r* = 0.62). Total tree height (TTH), DBH, and tree aboveground biomass (AGB) were also positively correlated with *D*_*b*_ (*r* = 0.34–0.47).

Linear regression models (Table 2) revealed that the combination of crown length (CL), crown volume (CV), and total tree height (TTH) explained 64% of the variation in *D*_*b*_ among the 546 trees (Model 1). Using mean crown radius (MCR) instead of crown volume (CV) (Model 2) decreased the explanation power by 3% and resulted in a notable increase in AIC (Akaike Information Criterion). In Models 1 and 2, total tree height had negative coefficient values, which signals an inverse effect on box‐dimension. This means that smaller trees with a larger crown length and greater volume or a larger mean crown radius exhibited higher structural complexity. Considering only mean crown radius (MCR) and crown base height (CBH) (Model 3) or crown length and height of maximum crown area (H_maxarea_) (Model 4) without using total tree height (TTH) reduced the explanation power by 4% compared to Model 1, with a distinct increase in AIC. In both of these models, CBH and H_maxarea_ have a negative influence on *D*_*b*_. Using simple linear regression, aboveground biomass explained only a small portion of the variation in *D*_*b*_ (*R* ^2^ = 0.22, AIC = 402.50, RMSE = 4.02).

**TABLE 2:** Best multiple linear regression models of tree structural complexity (box dimension *D*_*b*_) from combinations of architectural variables. Crown length (CL), crown volume (CV), total tree height (TTH), mean crown radius (MCR), crown base height (CBH), and height of maximum crown area (Hmaxarea). All listed variables had significant effects in the model (*p* < 0.05). Model performance was evaluated in terms of Akaike information criterion (AIC), adjusted *R* ^2^ and root mean square error (RMSE).

S.N.	Model	AIC	*R* ^2^	RMSE
1	*D*_*b*_ = 1.95 + 0.067*log (CL) + 0.155log (CV)−0.312*log (TTH)	−790.68	0.64	2.72
2	*D*_*b*_ = 2.12 + 0.176*log (CL) + 0.196*log (MCR)−0.274*log (TTH)	−742.72	0.61	2.83
3	*D*_*b*_ = 1.90 + 0.193*log (MCR)−0.088*log (CBH)	−728.77	0.60	2.88
4	*D*_*b*_ = 1.95 + 0.184*log (MCR) + 0.078*log (CL)−0.150*log (Hmaxarea)	−720.54	0.60	2.91

### 3.3 *D*_*b*_ and Tree Interaction

On our 14 plots, the stem density varied from 245 stems ha^−1^ ( *Alnus nepalensis* ) to 2242 stems ha^−1^ (*Pinus wallichiana*, pure). Species richness varied from one species (11 plots) to two species (3 plots). The three mixed plots had *Pinus wallichiana* , either with *Cupressus torulosa* (2 plots) or *Abies spectabilis* (1 plot). The Shannon Diversity Index (H) ranged from 0 to 0.61. A mixed‐effect model with the target variable *D*_*b*_, the fixed predictor variables H, species richness, and stem density, and plot as a random factor had a conditional *R* ^2^ of 0.17 and a marginal *R* ^2^ of 0.09 (Table 3). However, H and species richness were not significant in the model (Table A2). The best performing model (lowest AIC = −267.64) comprised stem density as a single fixed factor and plot as a random factor and had a conditional *R* ^2^ of 0.19 and a marginal *R* ^2^ of 0.08. The inclusion of a random variable to the fixed variable thus explained an additional 11% of the variance. The model indicates trees will have lower *D*_*b*_ at higher stem density plots (Table A3).

**TABLE 3:** Linear mixed‐effect models for the target variable box‐dimension (*D*_*b*_) (*n* = 546 trees). The fixed factors were Shannon Index (H), species richness (S), and stem density ha^−1^ (stem density) with plot as the random factor. The full model considered all fixed factors while the best model was chosen based on model performance (AIC). Model performance is evaluated in terms of the Akaike information criterion (AIC), conditional and marginal coefficients of determination (*R* ^2^), and root mean square error (RMSE).

Model		AIC	*R* ^2^ (cond.)	*R* ^2^ (marg.)	RMSE
Full model	*D*_*b*_ = H + S+ stem density + (1| Plot)	−259.60	0.17	0.09	0.1816
Best model	*D*_*b*_ = stem density + (1| Plot)	−267.64	0.19	0.08	0.1814

### 3.4 *D*_*b*_ and Environment

On our 14 plots, elevation varied from 1300 to 3400 m asl, mean annual precipitation 1180 to 3600 mm yr.^−1^, and slope from 2° to 40°. A mixed effects model with the target variable *D*_*b*_ and the fixed predictor variables elevation, precipitation, and slope, along with the plot as a random factor, had a conditional *R* ^2^ of 0.14 and a marginal *R* ^2^ of 0.09 (Table 4). However, in this model, elevation and slope were not significant (Table A4). The best model (lowest AIC = −272.97) comprised precipitation as a single fixed factor and plot as a random factor and had a conditional *R* ^2^ of 0.13 and marginal *R* ^2^ of 0.08. The inclusion of a random variable thus explained an additional 5% of variance. The model showed a slight trend where, with the increase in precipitation from one plot to another, the tree's *D*_*b*_ increased (Table A5).

**TABLE 4:** Linear mixed‐effect models for the target variable box‐dimension (*D*_*b*_) (*n* = 546 trees). The fixed factors were elevation (m), precipitation (mm yr^−1^), and slope (^o^) with plot as the random factor. The full model considered all fixed factors while the best model was chosen based on model performance (AIC). Model performance is evaluated in terms of the Akaike information criterion (AIC), conditional and marginal coefficient of determination (*R* ^2^), and root mean square error (RMSE).

Model		AIC	*R* ^2^ (cond.)	*R* ^2^ (marg.)	RMSE
Full model	*D*_*b*_ = elevation + precipitation + slope + (1| Plot)	−259.17	0.14	0.09	0.1818
Best model	*D*_*b*_ = precipitation + (1| Plot)	−272.97	0.13	0.08	0.1822

To examine species‐specific influences of environmental factors on *D*_*b*_ more closely, we chose Pinus, which was present in the most plots (*n* = 7 plots) and had the most individual trees (*n* = 357 trees). The structural complexity of Pinus trees showed a slight inverse relationship with elevation and slope and a positive relationship with precipitation, with a conditional *R* ^2^ of 0.06 and a marginal *R* ^2^ of 0.05.

Environmental factors were also found to influence total tree height, with a strong inverse relationship observed between tree height and elevation, with a conditional *R* ^2^ of 0.80 and a marginal *R* ^2^ of 0.35 (Table A6). However, precipitation and slope showed no significant effects.

**FIGURE 5:**
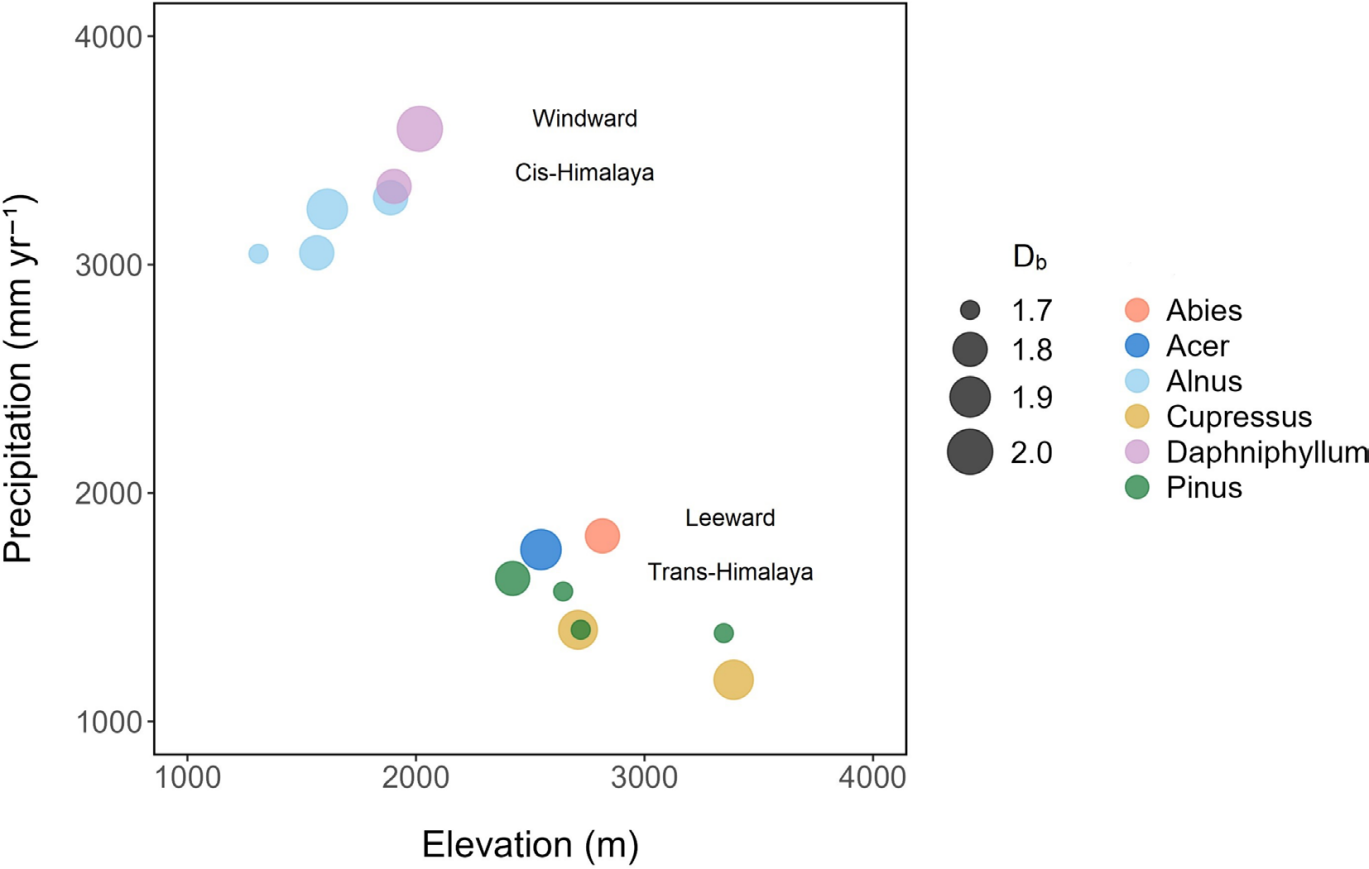
The study plots (*n* = 14) and their dominant tree species positioned along gradients of elevation and precipitation, wherein different dot colors indicate the different tree species and dot sizes indicate the respective mean box‐dimension (*D*_*b*_) values. Elevation data are from global digital elevation model (DEM) V003 (NASA/METI/AIST/Japan Spacesystems and U.S./Japan ASTER Science Team 2019) and precipitation data from CHELSA V2.1 (Karger et al. 2021).

At plot level, plots dominated by *Alnus nepalensis* and *Daphniphyllum himalense* in the Cis‐Himalaya region (Kaski and Lamjung) of the Annapurna Himal with high precipitation had *D*_*b*_ means between 1.7 and 2.0. Plots dominated by *Pinus wallichiana, Abies spectabilis, Cupressus torulosa*, and *Acer* spp. in the trans‐Himalayan region (Manang and Mustang) with low precipitation had *D*_*b*_ means between 1.7 and 1.9 (Figure 5).

### 3.5 *D*_*b*_ at the Tree and Stand‐Level

**FIGURE 6:**
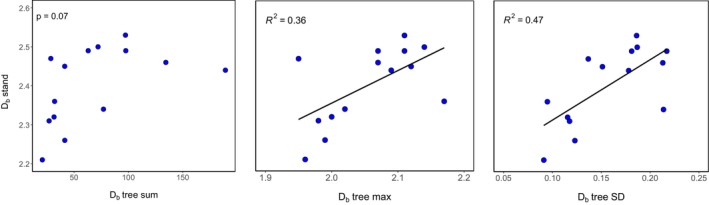
Relationship between box‐dimension (D_b_) at tree‐level and stand‐level (*n* = 14 plots), *p* < 0.05.

For our 14 plots, stand‐level *D*_*b*_ values by Basnet et al. (2025) ranged from 2.21 to 2.53. Our analysis revealed no significant correlation between the sum *D*_*b*_ of individual trees and the stand‐level *D*_*b*_ (*p* = 0.07) (Figure 6). The maximum tree‐level *D*_*b*_ of a plot explained 36% of the variation in stand‐level *D*_*b*_. The highest *R* ^2^ was obtained by the tree‐level standard deviation of *D*_*b*_ per plot (0.47).

## 4 Discussion

In the studied Himalayan forests, we found that tree‐level *D*_*b*_ was, to a large extent, explained by tree architecture, in particular crown characteristics, and to some extent by stem density and precipitation. The variation of tree‐level complexity was a strong predictor of stand‐level structural complexity.

Mean *D*_*b*_ per species varied between 1.89 (*Dahniphyllum himalenese*) and 1.70 ( *Pinus wallichiana* ). It was thus somewhat higher than for most studied European trees, including *Pinus sylvestris* (1.6 and 1.68); (Saarinen et al. 2021; Tienaho et al. 2022), *Fraxinus excelsior* (1.48 and 1.52), *Fagus sylvatica* (1.69 and 1.66), *Picea abies* (1.8) and *Acer pseudoplatanus* 1.65 (Seidel 2018; Seidel et al. 2019c). The reasons remain speculative and probably deserve more scientific attention, but the old‐growth and undisturbed characteristics of the studied Himalayan forests may be among them.

We found clear relationships between tree architecture and *D*_*b*_. In our multivariate models, combining total tree height, crown length, and crown volume explained 64% of the variation in *D*_*b*_ (Table 2). Our findings are consistent with Seidel et al. (2019a), who observed that in mixed forests comprising four broad‐leaved tree species, *D*_*b*_ was significantly related to the mean crown radius and crown surface area.

Our model revealed a negative influence of total tree height on *D*_*b*_, while crown structure positively influenced *D*_*b*_. As such, in *Alnus nepalensis*, structural complexity decreased with increasing height. Likewise, *Cupressus torulosa* maintained higher complexity at lower tree heights. This observation aligns with the findings in *Pinus sylvestris* and *Fagus sylvatica* in Central Europe (Seidel and Böttger 2023). Taller trees tend to prioritize vertical growth, allocating more resources to strengthening their trunks. In contrast, shorter trees focus more on branching and structural development, emphasizing lateral crown expansion, as stated by (McMahon and Kronauer 1976; Osada 2011). In some resource‐rich environments, taller trees may develop extensive branching and crown structures and thus exhibit higher complexity (Poorter et al. 2006). Conversely, taller trees with larger canopies may exhibit low structural complexity if their branching is uniform and branch angles are minimal (Pretzsch 2014). In our study, all taller tree species (*Pinus wallichiana, Abies spectabilis and Alnus nepalensis*) had crowns with low branching structure and thus relatively low structural complexity.

Larger crown structures were also found to have positive effects on the structural complexity of individual trees (Olivier et al. 2016; Reich et al. 2021) and crown structures generally had stronger relationships with *D*_*b*_ than stem attributes (i.e., tree height, DBH, and volume) (Saarinen et al. 2021). Models in our study also indicated that *D*_*b*_ decreased with increasing tree crown base height and increasing height of the maximum crown projection area. The increase in vertical crown structure could be attributed to the tree's simplified structure with fewer branches, potentially resulting from reduced light availability in the lower parts of the tree (Osada and Takeda 2003) or drought‐induced dieback of branches (Heidenreich and Seidel 2022). Our results underscore the critical role of horizontal crown structure, rather than vertical structure alone, in determining structural complexity.

We further observed that aboveground biomass only played a minor role in explaining variation in *D*_*b*_. This may be attributed to the fact that most biomass is concentrated in the stems, whereas structural complexity is primarily driven by a tree's spatial occupation (Seidel and Böttger 2023; Stiers et al. 2020).

The role of tree interaction was explored using mixed effect models; we found that stem density (ha^−1^) explained 19% of the variation in *D*_*b*_, wherein *D*_*b*_ decreased with increasing stem density. Similar to our findings, another study observed an increase in *D*_*b*_ from an average value of 1.5 to 1.7 following the thinning procedure of *Pinus sylvestris* , which lowered stem density (Saarinen et al. 2021). While no thinning had been applied in the stands in our study, we found indications that, also under near‐natural conditions, the *D*_*b*_ of an individual tree decreases with increasing stem density. This can be expected, as denser growth triggers height growth and an upward shift of the crown base height, with a longer branch‐free length of the bole and reduced complexity as a consequence.

Looking at the influence of environmental variables in determining *D*_*b*_, we found *D*_*b*_ increased with increasing precipitation, but elevation and the steepness of the slope had no influence. Our mixed‐effect model (Table 4) showed the best model with precipitation explaining 13% of the variation in *D*_*b*_. The effect of precipitation follows our expectations and was also found in previous, somewhat similar studies (Ehbrecht et al. 2021; Soto et al. 2024); however, the absence of an effect of elevation was unexpected. Tree height did decrease with increasing elevation, but small trees often had relatively high *D*_*b*_. Therefore, the change in *D*_*b*_ with elevation was less pronounced than the decline in tree height. This aligns with the findings on stand‐level complexity across 69 plots in the aforementioned study (Basnet et al. 2025), where environmental factors played a minor role in explaining stand‐level *D*_*b*_.

Overall, we found that species interactions and environmental variables had only limited ability to explain variations in *D*_*b*_. The observed findings may be attributed to the fact that most of our plots were composed of pure stands, limiting the availability of the data to discern clear patterns of species interaction effects. There may also be other unaccounted ecological factors explaining *D*_*b*_, e.g., wind (Dorji et al. 2021), light availability among the trees, differences in tree height, growth, crown asymmetry (Chen et al. 2014; Seidel et al. 2019c) and competition between trees, which decreases *D*_*b*_ (Seidel et al. 2011; Van de Peer et al. 2017). Similarly, soil properties (soil depth, moisture and nutrient content) influence tree growth and structure (Jobbágy and Jackson 2001); tree vitality influences complexity (Heidenreich and Seidel 2022); and forest age or forest at a successional stage induce differences in forest structure (Matsuo et al. 2021) and structural complexity (Stiers et al. 2018).

One should also consider the special environment in the Annapurna region. Precipitation increases with elevation on the southern windward side of the Annapurna Himal and decreases on its northern leeward side. Likely these factors interact in a way that tends to level off more pronounced effects. Furthermore, it needs to be pointed out that elevation is only a proxy variable, with more complex underlying changes in the related environmental factors such as temperature, air pressure, and atmospheric composition. Our study covered an elevation range from 1300 m to 3400 m asl, with the treeline being located around 4200 m a.s.l (Shrestha et al. 2007), (Figure 1). Changes in tree structure are likely to become more pronounced if the treeline ecotone is also considered. At the species level, we mainly observed that the *D*_*b*_ of *Pinus wallichiana* trees decreased with increasing elevation and slope but increased with higher precipitation levels. However, the explained variation was rather low. Although not directly testable for all species with our data set, our findings indicate a primary role of turnover in species with environmental factors in these Himalayan forests, resulting in changes in tree structural complexity (Figure 5). To test this contention, data from more plots, trees, and tree species would be needed. Definitely, also high resolution and temporally aligned data on micrometeorological variables would improve such analyses. It could be that today's forest structures are already partly decoupled from climate that was of decades ago. In addition, the used broad interpolations may not fully cover the actual climatic variability.

Our final hypothesis addressed the relationship between tree‐level and stand‐level complexity. We found that the standard deviation of tree‐level *D*_*b*_ within a plot was the strongest predictor of stand‐level *D*_*b*_ (*R* ^2^ = 0.47), followed by the maximum tree‐level *D*_*b*_ (*R* ^2^ = 0.36) (Figure 6). This is somewhat different from the only other study available in this direction, from a temperate German broad‐leaved forest. There, maximum tree‐level *D*_*b*_ was the strongest predictor, whereas standard deviation played a minor role. There are many differences between trees and stands in the Himalaya and in Central Germany, which renders it almost impossible to distill possible reasons for the difference. It can be concluded that both variability and maximum values in tree‐level *D*_*b*_ are important drivers of stand‐level *D*_*b*_.

### 4.1 Considerations for Nature Conservation and Forest Management

Himalayan forests are under threat by climate change (Negi et al. 2022) and one has to expect changes in forest structure and communities. Here, we provide needed baseline information regarding tree and forest structures. The observed minor role of environmental factors would probably mean that smaller changes in climate will not change much in forest structure; it was, however, discussed that today's forest structures could already have decoupled from the climate in the past and that the available broad interpolations may not cover the actual climatic variability. Another striking change in the forest regions of the Himalaya concerns socio‐economic dynamics (McGunnigle et al. 2025). Around tourist centers in the Himalaya region, human interference by the use of forest products may rise; on the other hand, the region experiences significant outmigration with the abandonment of agricultural fields. Also in this respect, we provide baseline data regarding potential forest degradation but also successional development. In terms of forest use, “management for complexity” is ranking high in western silviculture (e.g., Messier et al. 2013). Our data would suggest that in the Himalayan Forest, first of all, the variability among trees should be maintained, and in addition, the most complex individual trees should remain in the stand. Given that the region under study is located within a conservation area, this research highlights the significance of complex trees in enhancing habitat quality and fostering biodiversity conservation.

## Author Contributions

**Smita Das:** conceptualization (equal), data curation (lead), formal analysis (lead), funding acquisition (lead), investigation (lead), methodology (lead), software (equal), validation (lead), visualization (lead), writing – original draft (lead), writing – review and editing (equal). **Prakash Basnet:** data curation (supporting), investigation (equal), methodology (equal), software (supporting), visualization (supporting), writing – review and editing (supporting). **Dominik Seidel:** conceptualization (equal), data curation (supporting), formal analysis (supporting), methodology (supporting), software (equal), supervision (supporting), visualization (supporting), writing – review and editing (supporting). **Alexander Röll:** methodology (supporting), project administration (supporting), visualization (supporting), writing – review and editing (equal). **Martin Ehbrecht:** visualization (supporting), writing – review and editing (equal). **Dirk Hölscher:** conceptualization (lead), formal analysis (equal), investigation (equal), methodology (equal), project administration (supporting), supervision (lead), visualization (equal), writing – original draft (supporting), writing – review and editing (equal).

## Conflicts of Interest

The authors declare no conflicts of interest.

## Data Availability

The raw data used in the study are available at https://doi.org/10.25625/KBA1KN.

## Appendix A

**TABLE A1:** Climatic and topographic characteristics and diversity of tree species of the study plots (*n* = 14). Climate data were extracted from CHELSA time series data version 2.1 (1981–2010, (Karger et al. 2021), elevation and aspect data from (NASA/METI/AIST/Japan Spacesystems and U.S./Japan ASTER Science Team 2019), and the slope was measured with an Abney level in the field. The Shannon Diversity Index was calculated based on the field assessment of tree species present in each plot.

	Min	Max	Mean ± SD
Precipitation (mm year ^−1^)	1182	3595	2265 ± 919
Average annual temperature (°C)	2.4	17.2	10.2 ± 4.7
Elevation (m)	1311	3390	2350 ± 648
Slope (^o^)	2	40	15 ± 12
Aspect (northness)	‐1	1	0.21 ± 0.8
Aspect (eastness)	−0.9	0.9	0.02 ± 0.6
Shannon Diversity Index	0	1.8	0.58 ± 0.56

**TABLE A2:** Full model estimates and statistics of the linear mixed effect model for the target variable *D*_*b*_ with Shannon Index (H), species richness, and stem density ha^−1^. Estimates are provided with standard errors (SE), confidence intervals (CI), and significance levels (*p*). All fixed factors are scaled, and the plot is considered random.

Predictors	*Db*		
	Estimates	CI	*p*
(Intercept)	1.75	1.71 to 1.79	**< 0.001**
H	0.06	−0.11 to 0.22	0.471
Species richness	−0.10	−0.26 to 0.06	0.226
Stem density	−0.05	−0.09 to −0.01	**0.007**
Random effects			
*σ* ^2^	0.03		
τ_00 plot_	0		
ICC	0.09		
N _plot_	14		
Observations	546		
Marginal *R* ^2^/conditional *R* ^2^	0.094/0.174		

*Note:* A bold *p*‐value indicates statistically significant effect at *p* < 0.05.

**TABLE A3:** Best model estimates and statistics of the linear mixed effect model for the target variable *D*_*b*_ with predictor variable stem density ha^−1^. Estimates are provided with standard errors (SE), confidence intervals (CI), and significance levels (*p*).

Predictors	*Db*		
	Estimates	CI	*p*
(Intercept)	1.75	1.70 to 1.79	**< 0.001**
Stem density	−0.06	−0.10 to −0.01	**0.016**
Random Effects			
*σ* ^2^	0.03		
τ_00 plot_	0.00		
ICC	0.13		
N _plot_	14		
Observations	546		
Marginal *R* ^2^/conditional *R* ^2^	0.078/0.19		

*Note:* A bold *p*‐value indicates statistically significant effect at *p* < 0.05.

**TABLE A4:** Full model estimates and statistics of the linear mixed effect model for the target variable *D*_*b*_ with predictor as environmental variables. Estimates are provided with standard errors (SE), confidence intervals (CI), and significance levels (*p*). All fixed factors are scaled, and the plot is considered random.

Predictors	*Db*		
	Estimates	CI	*p*
(Intercept)	1.75	1.72 to 1.78	**< 0.001**
Precipitation	0.07	0.02 to 0.13	**0.013**
Elevation	0.01	−0.05 to 0.07	0.708
Slope	−0.02	−0.05 to 0.01	0.173
Random effects			
*σ* ^2^	0.03		
τ_00 plot_	0		
ICC	0.06		
N _plot_	14		
Observations	546		
Marginal *R* ^2^/conditional *R* ^2^	0.090/0.148		

*Note:* A bold *p*‐value indicates statistically significant effect at *p* < 0.05.

**TABLE A5:** Best model estimates and statistics of the linear mixed effect model for the target variable *D*_*b*_ with predictor variable precipitation. Estimates are provided with standard errors (SE), confidence intervals (CI), and significance levels (*p*).

Predictors	*Db*		
	Estimates	CI	*p*
(Intercept)	1.75	1.72 to 1.78	**< 0.001**
Precipitation	0.06	0.03 to 0.08	**< 0.001**
Random effects			
*σ* ^2^	0.03		
τ_00 plot_	0		
ICC	0.05		
N _plot_	14		
Observations	546		
Marginal *R* ^2^/conditional *R* ^2^	0.089/0.137		

*Note:* A bold *p*‐value indicates statistically significant effect at *p* < 0.05.

**TABLE A6:** Best model estimates and statistics of the linear mixed effect model for the target variable total tree height with predictor variable elevation. Estimates are provided with standard errors (SE), confidence intervals (CI), and significance levels (*p*).

Predictors	Total tree height		
	Estimates	CI	*p*
(Intercept)	14.13	11.45–16.82	**< 0.001**
Elevation	−4.19	−6.37 to −2.01	**< 0.001**
Random effects			
*σ* ^2^	10.16		
τ_00 plot_	21.38		
ICC	0.68		
N _plot_	14		
Observations	546		
Marginal *R* ^2^/conditional *R* ^2^	0.358/0.793		

*Note:* A bold *p*‐value indicates statistically significant effect at *p* < 0.05.
